# Idiopathic retinal vasculitis, arteriolar macroaneurysms and neuroretinitis: clinical course and treatment

**DOI:** 10.1186/1869-5760-3-21

**Published:** 2013-01-25

**Authors:** Alexander Rouvas, Eleni Nikita, Nikos Markomichelakis, Panagiotis Theodossiadis, Nikolaos Pharmakakis

**Affiliations:** 1 2nd Ophthalmology Department, Attikon University Hospital, Athens, 12462, Greece; 2 Manchester Royal Eye Hospital, Manchester, M13 9WH, UK; 3 Ocular Immunology and Inflammation Clinic, 1st Ophthalmology Department, G. Gennimatas University of Athens, Athens, 12462, Greece; 4 University of Athens, Athens, 12462, Greece; 5 Department of Ophthalmology, University of Patras Hospital, Patras, 26504, Greece

**Keywords:** Inflammation, Retinal disease, Vasculitis

## Abstract

**Background:**

The purpose of the study is to describe the clinical course and treatment of idiopathic retinitis, vasculitis, aneurysms and neuroretinitis. The study utilized non-randomized, retrospective and interventional case series. The eight eyes of six patients were analysed. Testing included wide fluorescein angiography, indocyanine green angiography and systemic evaluation. Treatment involved observation, panretinal laser photocoagulation (PRP) for peripheral retinal ischemia, grid laser for macular oedema and focal laser on the macroaneurysms. The main outcome measures were initial visual acuity (VA), initial stage at diagnosis, clinical course, surgical intervention, final VA, final stage and complications of disease.

**Results:**

Five out of eight eyes with retinal ischemia in more than two quadrants that were treated with PRP and grid laser for macular oedema maintained excellent VA and demonstrated no progression of retinal ischemia during follow-up. The two eyes which exhibited retinal ischemia in less than two quadrants and macular oedema were treated with grid laser and focal laser on the macroaneurysms, but did not undergo PRP. VA improved by two lines of the Snellen chart, and there was no progression of retinal ischemia during the 3 and 4 years of follow-up. One eye with neither retinal ischemia nor macular oedema was not treated, and the clinical picture remained stable during the follow-up.

**Conclusion:**

Early PRP may be considered in the presence of angiographic evidence of peripheral retinal non-perfusion. However, treatment could be withheld until the patient develops retinal ischemia in more than two quadrants.

## Background

Idiopathic retinal vasculitis, arteriolar macroaneurysms and neuroretinitis (IRVAN) is a rare but well-defined clinical entity. Patients are usually young, with an average age of 30 years old at presentation, with female predominance and no associated systemic illness. The disorder may present with exudation, which is caused by the macroaneurysms, and vascular sheathing. The macroaneurysms of IRVAN may present throughout the fundus but are more common on the larger retinal arterioles, typically on the bifurcations. They have the tendency to leak with lipid deposition which can lead to visual loss if there is extension towards the macula. Peripheral capillary non-perfusion is another main predisposing factor of vision loss and a prominent feature of the syndrome. It may progress posteriorly and lead to pre-retinal neovascularization and secondary vasoproliferative complications [1,2].

Management of IRVAN reveals a variety of treatment suggestions. The therapeutic regimens advocated include panretinal laser photocoagulation (PRP), macular grid photocoagulation, surgery, transcleral cryotherapy, steroids therapy and administration of monoclonal antibodies such as ranibizumab and infliximab [2-4].

Samuel et al. conducted the largest cohort study of IRVAN patients and concluded that PRP should be considered in the presence of retinal ischemia, irrelevant to the extent of retinal non-perfusion, in order to prevent the development of ocular neovascularisation and to ensure a good long-term visual outcome [5]. In our study PRP application for each patient was individualised according to the degree of retinal ischemia, and a staging system is proposed to quantify retinal ischemia (Table 1).

**Table 1:** Staging of the ocular findings in idiopathic retinal vasculitis, arteriolar macroaneurysms and neuroretinitis

**Stage**	**Ocular findings**
1	Macroaneurysms, exudation, neuroretinitis, retinal vasculitis
2	Capillary non-perfusion (angiographic evidence)
3	Posterior segment neovascularization of disc or elsewhere and/or vitreous haemorrhage
4	Anterior segment neovascularization (rubeosis iridis)
5	Neovascular glaucoma

## Results

Demographic information, clinical data and the type of treatment are listed in the Table 2. A total of eight eyes of six patients were studied retrospectively. Two patients had bilateral disease (patient numbers 3 and 4). Average age at the time of diagnosis was 47 years (range, 34 to 80 months). There was female predominance by 5:1. All patients were Caucasian. The average length of follow-up was 25.6 months (range, 19 to 48 months). Two eyes had retinal ischemia in less than two quadrants (patient numbers 5 and 6) and five eyes in more than two quadrants (cases 1, 3 and 4). All reached stage 2 of the disease at the last follow-up, apart from one eye which was classified as stage 1 at the time of diagnosis (patient number 1). The clinical course of each eye after the initiation of treatment was evaluated with respect to the final VA and the stage of ischemic retinopathy at the initiation of treatment.

**Table 2:** Demographic information, clinical data and the type of treatment of patients

**Patient**	**Age/gender**	**Cases**	**Eye**	**Initial BCVA**	**Final BCVA**	**Initial CFT**	**Final CFT**	**Initial stage**	**Final stage**	**PRP**	**Grid laser**	**Focal laser**	**Follow-up (months)**
1	36/F	1	Right	9/10	9/10	275	228	2	2	Yes	Yes	No	24
2	47/F	2	Left	10/10	10/10	236	221	1	1	No	No	No	7
3	34/F	3	Right	8/10	9/10	218	228	2	2	Yes	No	No	24
		4	Left	08/10	09/10	220	225	2	2	Yes	No	No	24
4	38/M	5	Right	10/10	10/10	230	227	1	2	Yes	No	No	18
		6	Left	10/10	10/10	221	218	1	2	Yes	No	No	18
5	80/F	7	Right	3/10	5/10	285	225	2	2	No	Yes	Yes	24
6	48/F	8	Left	7/10	9/10	267	218	2	2	No	Yes	Yes	36

*BCVA* best corrected visual acuity; *CFT* central foveal thickness; *PRP* panretinal photocoagulation; *F* female; *M* male.

**Figure 1:**
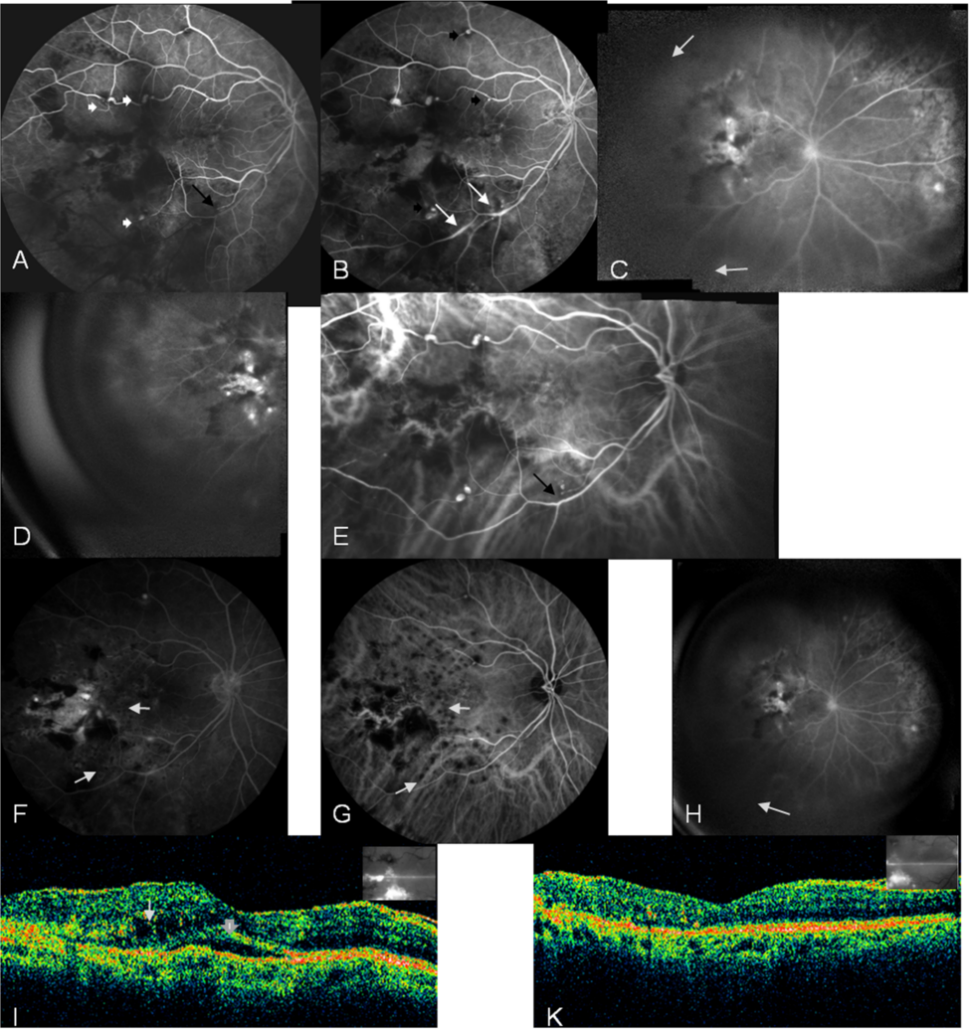
**Arteriovenous phase fluorescein angiogram of retinal arterioles.** (**A**) Arteriovenous phase fluorescein angiogram (FA) showing multiple aneurysmal dilatations along the upper and lower temporal retinal arterioles (white arrow heads) as well as the abrupt obstruction of the lower temporal arterial branch (black arrow). (**B**) FA at 2.5 min demonstrating multiple aneurysms along bifurcations of retinal arcades (black arrow heads) and vasculitis along the lower temporal arterial arcade (white arrows). (**C**, **D**) Wide FA displaying retinal ischemia in less than two quadrants (area between the white arrows) and a hot disc. (**E**) Indocyanine green angiogram (ICG) illustrating multiple aneurysmal dilatations along the upper and lower temporal retinal arterioles and the abrupt obstruction of the lower temporal arterial branch (black arrow). (**F**, **G**) Late phase FA and mid-phase ICG 12 months post-treatment demonstrating the area of grid laser photocoagulation (white arrows), and the obstruction of the arterial branch peripheral to the focal laser (black arrow) with cessation of leakage. (**H**) Wide FA 36 months post-treatment showing no progression of retinal ischemia to more than two quadrants. (**I**) OCT pre-treatment showing intraretinal (white arrow) and subretinal fluid (white arrowhead). (**K**) OCT post-treatment demonstrating resolution of macular oedema.

**Figure 2:**
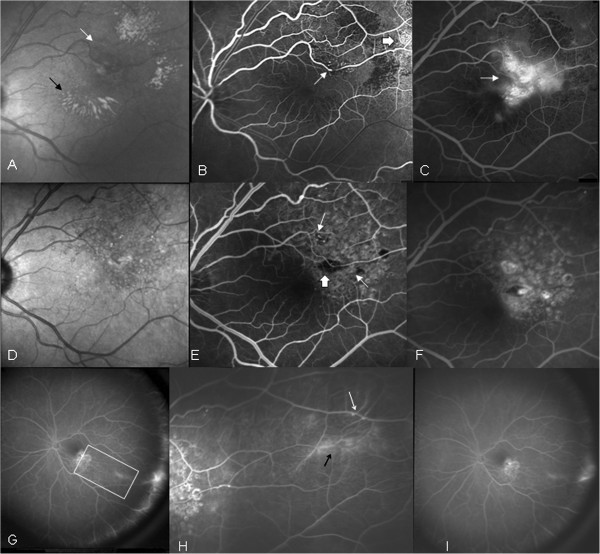
**Infrared fundus photograph of left eye.** (**A**) Infrared fundus photograph of left eye showing blood (white arrow) and hard exudates (black arrow). (**B**) Arteriovenous phase FA showing macroaneurysms along bifurcations of retinal arcades (white arrow) as well as vasculitis of retinal arterioles (black arrow). (**C**) Late phase FA demonstrating leakage from the macroaneurysms and telangiectatic retinal net (white arrow). (**D**) Infrared fundus photograph 12 months post-treatment showing no hard exudates or blood. (**E**) Arteriovenous phase FA 12 months post-treatment demonstrating the area of grid laser photocoagulation (white arrows) as well as the focal laser at the macroaneurysm (black arrow). (**F**) Late phase FA 12 months post-treatment showing no dye leakage. (**G)** Wide FA 12 months post-treatment displaying ischemia in less than two quadrants of the retina. (**H**) Magnified triangular area of retinal ischemia of the previous picture on wide FA with aneurismal dilatations along retinal bifurcations (white arrows) and vasculitis (black arrow). (**I**) Wide FA 48 months post-treatment showing no progression of ischemia.

Five out of eight eyes with ischemia in more than two quadrants were treated with PRP (cases 1, 3, 4, 5 and 6), and one of these eyes also underwent grid laser (case 1). They all maintained excellent VA at the end of follow-up period and demonstrated no progression of the retinal ischemia. The two out of eight eyes at stage 2 (cases 7 and 8), which exhibited retinal ischemia in less than two quadrants, were treated only with grid laser on the macular area of leakage and focal laser on the macroaneurysms (Figures 1F, G and 2E). At the end of follow-up period, the VA improved by two lines of the Snellen chart, and there was no progression of retinal ischemia during the 3 and 4 years of follow-up respectively. The last one eye of stage 1 was not treated, and the clinical picture remained stable during the 19 months of follow-up. Among the three eyes with macular oedema that were treated with grid laser (cases 1, 7, 8), the mean central foveal thickness (CFT) measured by optical coherence tomography (OCT) at initial visit was 276 μm (range, 267 to 285 μm) and at final visit was 224 μm (range, 218 to 228 μm) (Table 2). Written informed consents (in Greek) were obtained from the patient for publication of this article and accompanying images.

## Discussion

There is little firm information on the natural history and visual outcome of the disease in literature. All of the reported cases have limitations, such as small sample size, adopting therapeutic regimens that have limited scientific validity, narrow period of follow-up and personal biases. Several features have been reported about IRVAN, including an association with fungal sinusitis, initial presentation as optic disc oedema with elevated intracranial hypertension and a rapid change in the dynamics of the aneurysms [6-8].

Over the years, many therapeutic regimens have been evaluated. The main treatments can be divided into three categories: (1) medical, (2) surgical and (3) photocoagulation which includes PRP, focal laser on leaking macroaneurysms and grid laser photocoagulation for macular oedema.

The presence of the anterior chamber and vitreous cells as well as the rapid change in the morphological appearance and location of the aneurysms could be the result of a migratory inflammatory process involving the alternate segments along the vascular tree [9]. However, therapeutic response to steroids is absent or minimal [1,2,5,8,10]. In the quest of better alternatives to steroids, antitumor necrosis factor agent therapy has been appraised with promising results [4].

Vitrectomy has been used in the presence of vitreous haemorrhage and in conjunction to PRP in an attempt to improve visual outcome [5]. Recently, a case report has been published regarding the use of intravitreal ranibizumab treatment combined with PRP and vitrectomy [3].

PRP has been suggested as the treatment of choice for IRVAN in the presence of widespread retinal ischemia so as to prevent the development of ocular neovascularization and vitreous haemorrhage [5]. The rationale for this therapeutic approach was based on the results of the Diabetic Retinopathy Study and the Central Vein Occlusion (CRVO) Study, which recommended argon laser PRP in high risk proliferative diabetic retinopathy and CRVO with iris neovascularisation respectively [11,12]. According to both studies, the presence of retinal or iris neovascularisation was a prerequisite for the initiation of PRP. However, Samuel et al. state that visual prognosis may depend on the early initiation of treatment with PRP for extensive ischemic retinal disease, even without the presence of neovascularization [5]. The extent of retinal non-perfusion though was not quantified.

In our case series, all eyes that were treated with PRP, irrelevant to the extent of retinal non-perfusion, maintained excellent VA and demonstrated no progression of retinal ischemia. Two eyes at stage 2, which exhibited retinal ischemia in less than two quadrants, were treated only with grid laser for macular oedema and focal laser on the macroaneurysms. VA improved by two lines of the Snellen chart, and there was no progression of retinal ischemia during the 3 and 4 years of follow-up period of these eyes (Figures 1H and 2I). One eye of stage 1 was not treated and the clinical picture remained stable.

Exudative maculopathy was responsible for the initial decrease in vision in the seven eyes of the review by Samuel et al. and in the five eyes of our patients. After application of grid/focal photocoagulation, two thirds of the eyes of the review maintained a VA within one line of the initial VA and all of our patients improved their VA by one or two Snellen lines. Direct photocoagulation to leaking macroaneurysms has been opposed due to the occlusion of the blood vessel originating at the site of the vessel burn [4,13]. However, laser was applied on the leaking macroaneurysms temporal to the fovea. Subsequent obstruction of the arterial branch in one eye did not affect VA and successfully prevented the leaking of the dye in FA (Figure 1F, G).

## Conclusions

Our results along with the authors suggest that the application of PRP may be useful in the treatment of patients with IRVAN. However, as the root of the disease is unknown, treatment does not eliminate the cause but aims to reduce or eliminate macular oedema, prevent long-term macular changes and the destructive consequences of retinal ischemia. The precise time to initiate treatment is controversial; as in opposition to other ischemic retinopathies, there have been no randomized prospective clinical trials.

In our patients, deferral of PRP when peripheral ischemia is present in less than two quadrants of the retina did not appear to result in progression of the ischemia in a follow-up period of 3 and 4 years for the two of the six of our patients. Therefore, treatment could be withheld until the patient develops retinal ischemia in more than two quadrants. For these circumstances wide fluorescein angiography (WFA) can be a useful diagnostic tool. Randomized studies with higher numbers of patients and long follow-up periods are required to demonstrate the benefits and efficacy of PRP in the management of IRVAN as well as its role in preventing recurrences.

## Methods

We retrospectively evaluated the medical records, fundus photographs and investigative studies of six patients who presented to our clinic with IRVAN within the last 5 years. The diagnostic studies included WFA and ICG with Heidelberg retinal angiography-2 (HRA-2, Heidelberg Engineering, Heidelberg, Germany) and OCT (Carl Zeiss, Meditec, Jena, Germany). Tests were initially performed at 3-month intervals for 1 year and at 6-month intervals for the following years, in order to appraise the clinical course of the disease and the efficacy of each treatment regimen. In view of the above tests, we also adopted a proposed classification scheme for staging retinal ischemia in IRVAN as proposed by Samuel et al. (Table 1). This classification has been proposed for other diseases such as central retinal vein occlusion and diabetic retinopathy. Additionally though, we implemented an advanced classification for stage 2 and onwards, where capillary non-perfusion was evaluated separately for each of four quadrants of the retina, based on WFA findings with the Straurenghi 230 scanning laser ophthalmoscope retina lens (Ocular Instruments, Glenfield, Auckland).

## Competing interest

The authors declare that they have no competing interests.

## Authors’ contributions

AR and EN conceived the study. AR, EN, NM, PT and NP gathered the data. EN analyzed the data. AR and EN wrote the article. AR, NM and NP edited the article. All authors read and approved the final manuscript.
